# Binding-and-Folding Recognition of an Intrinsically Disordered Protein Using Online Learning Molecular Dynamics

**DOI:** 10.1021/acs.jctc.3c00008

**Published:** 2023-06-21

**Authors:** Pablo Herrera-Nieto, Adrià Pérez, Gianni De Fabritiis

**Affiliations:** † Computational Science Laboratory, Universitat Pompeu Fabra, Barcelona Biomedical Research Park (PRBB), C Dr. Aiguader 88, 08003, Barcelona, Spain; ‡ Acellera Labs, C Dr Trueta 183, 08005, Barcelona, Spain; ¶ Acellera Ltd, Devonshire House 582, Stanmore Middlesex, HA7 1JS, United Kingdom; § Institució Catalana de Recerca i Estudis Avançats (ICREA), Passeig Lluis Companys 23, 08010 Barcelona, Spain

## Abstract

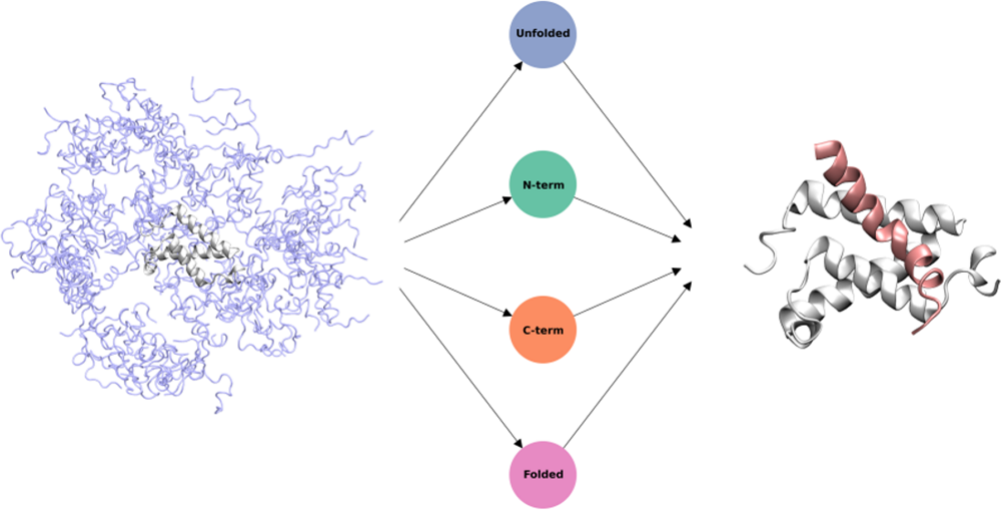

Intrinsically disordered proteins participate in many biological processes by folding upon binding to other proteins. However, coupled folding and binding processes are not well understood from an atomistic point of view. One of the main questions is whether folding occurs prior to or after binding. Here we use a novel, unbiased, high-throughput adaptive sampling approach to reconstruct the binding and folding between the disordered transactivation domain of c-Myb and the KIX domain of the CREB-binding protein. The reconstructed long-term dynamical process highlights the binding of a short stretch of amino acids on c-Myb as a folded α-helix. Leucine residues, especially Leu298-Leu302, establish initial native contacts that prime the binding and folding of the rest of the peptide, with a mixture of conformational selection on the N-terminal region with an induced fit of the C-terminal.

## Introduction

Intrinsically disordered proteins (IDPs) participate in many biological functions despite lacking a stable tertiary structure.^1^ Initial clues for the function of IDPs were revealed by structural studies,^2,3^ showing that proteins that were disordered in isolation became folded upon interacting with their partners, opening to question how folding couples with binding.

Recently, molecular dynamics (MD) simulations have been successfully applied to reconstruct biological dynamic events in problems such as protein–ligand^4^ and protein–protein^5,6^ binding, as well as protein folding.^7,8^ MD has also been applied successfully in the field of IDPs. Many studies have used MD simulations for general characterization of IDP dynamic properties and coupled binding and folding processes.^9−15^ In particular, the Mdm2 protein and the disordered 12-residue N-terminal region of p53 have been extensively studied using molecular dynamics, either by implicit solvent simulations,^16^ parallel full-atom simulations totaling,^17^ biased free-energy-based sampling,^18^ biased/unbiased simulations to estimate kinetics on the second time scale,^19^ and biased exchange metadynamics.^20^

Explicit solvent unbiased MD simulations have been successfully applied to study complete coupled binding and folding events for various systems.^21−23^ Most of these studies show a general tendency of IDPs to follow an induced-fit mechanism, although each system has its own singularities. The long 200 μs trajectory of the XD domain and N_TAIL_ performed in ref (23), which captured 72 binding and unbinding events, shows an interesting characteristic of the IDP of favoring unfolded states upon binding the XD domain, where partially folded states unfold to progress toward the final bound complex, and suggest that disordered states may be favored due to their kinetic advantage over folded states.

**Figure 1:**
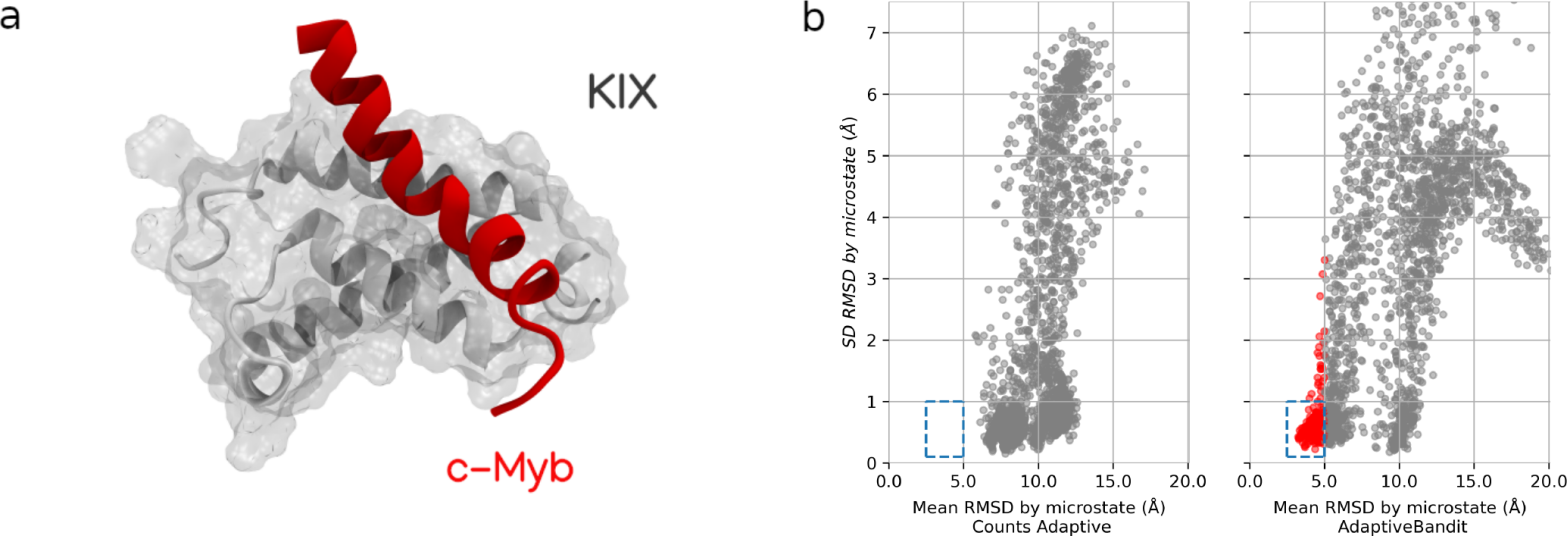
Exploration performance. (a) KIX—cMyb NMR structure. KIX domain is shown as a white surface and ribbon and c-Myb bound to KIX as a red helix (PDB code 1SB0). Exploration performance by (b) Counts Adaptive (∼480 μs) and AdaptiveBandit (∼450 μs) is shown by plotting the mean RMSD (on the *x*-axis) and standard deviation (on the *y*-axis) for each of the MSM’s microstates; all the states with a mean RMSD lower than 5 Å are colored in red. The dashed square indicates the *bound zone*, placed in the region corresponding to a low mean RMSD and standard deviation.

The KIX—c-Myb binding-and-folding mechanism has been extensively studied experimentally as an exemplar case of protein-IDP interaction.^24−30^ The KIX domain of the CREB-binding protein is a short 87-aa region composed of three α-helices (designated as α-1, α-2 and α-3, from N-terminal to C-terminal) forming a compact bundle.^3^ KIX represents a paradigm of binding promiscuity: it binds to many IDPs, including the proto-oncogene c-Myb^3^ (Figure 1.a), with multiple binding conformations.^24^ However, the system composed by KIX—c-Myb remained outside of the scope of all-atom molecular simulations due to the size of the IDP (it doubles the length of p53) and the existence of multiple binding modes between them.^24^ In particular, it is unclear whether the interaction takes place by conformational selection, i.e., c-Myb needs to be folded before binding to its partner, or by induced-fit, where binding not only happens independently of c-Myb’s secondary structure but also triggers its folding, as shown for other IDPs (KIX-pKID).^22,31^ Understanding these aspects has implications for the druggability of disordered proteins. Another important factor is c-Myb’s high helicity in isolation and the consequences it might exert on the final complex structure, which features an extended α-helical c-Myb bound to KIX. Some reports support the induced-fit approach based on kinetics and mutagenesis studies,^25,26,30^ while others advocate for a mixed mechanism;^24^ yet not a detailed model for the binding process is available.

In this paper, we take advantage of a novel algorithm that frames the MD sampling problem from a reinforcement learning perspective (see ref (32) and Methods) to reconstruct multiple binding modes between c-Myb and KIX. This sampling algorithm was key for us to reconstruct the binding process, as our previous attempts over the years using other state-of-the-art adaptive sampling methods^33,34^ were not successful, always failing to recover the NMR bound structure (Figure 1b). Results provide insights into the binding mechanism between these two proteins, supporting a mixed model that combines both conformational selection and an induced fit.

## Results and Discussion

### Adaptive Sampling the KIX—c-Myb Binding-and-Folding Process

Simulations to reconstruct the KIX—c-Myb binding mode were performed by following an adaptive sampling strategy. In adaptive sampling, successive rounds of simulations are performed in an iterative stepwise manner, where an acquisition function over the currently sampled conformations is defined. We compare two of the acquisition functions used for KIX—c-Myb simulations: a count-based one and another one inspired by reinforcement learning, part of the novel AdaptiveBandit method.^32^

The new AdaptiveBandit method is framed into a simplified reinforcement learning problem, the multiarmed bandit problem (see Methods). We use the upper confidence bound (UCB) algorithm^35^ to optimize an action-picking policy in order to maximize future rewards, optimally balancing the exploration of new higher rewarding actions with the exploitation of the most known rewarding ones. The reward function, which associates the action with the reward given by the system, defines what we want to optimize. In this work, we choose the reward to be minus the free energy of each configuration visited in the trajectory spawn from a given action (see eq 2 in Methods), where the free energy of a conformation is given by the corresponding Markov state model (MSM) microstate computed with the data available at the current sampling epoch.

Standard low counts adaptive sampling^33^ (hereby named Counts Adaptive) can be shown to be optimal in pure exploration conditions.^34^ Counts are computed over clusters of conformations; this method is, however, noisy, as clusters can be poorly populated. Therefore, in the implementation available in ref (34), counts are computed over a smaller subset by grouping clusters (microstates) into macrostates, constructing a Markov State Model (MSM)^36^ with the available data at each round. The acquisition function is given by proportionally choosing macrostates as 1/*c*, where *c* represents macrostate counts and by randomly selecting conformations within them.

A comparison between Counts Adaptive and AdaptiveBandit is provided in Figure 1.b. The batch based on counts failed to connect microstates similar to the NMR structure in over ∼480 μs, reaching at best an RMSD around 7 Å. For us, it was impossible to build an MSM with the bound state with previous methods, and novel approaches were needed to reconstruct the binding-and-folding process between KIX and c-Myb successfully. AdaptiveBandit provides converged estimates of kinetics and thermodynamics after just 150 μs of sampling (Figure S5). The information on the bound structure is never used in the sampling process. AdaptiveBandit is agnostic to any specific conformations, as it only uses the contact matrix between KIX and cMyb and cMyb dihedra as the features for each MSM constructed at every epoch. The information on the bound structure is only used in the MSM analysis after all simulations are done to better show how well the simulations capture the NMR structure.

### Identification of the Bound State

**Figure 2:**
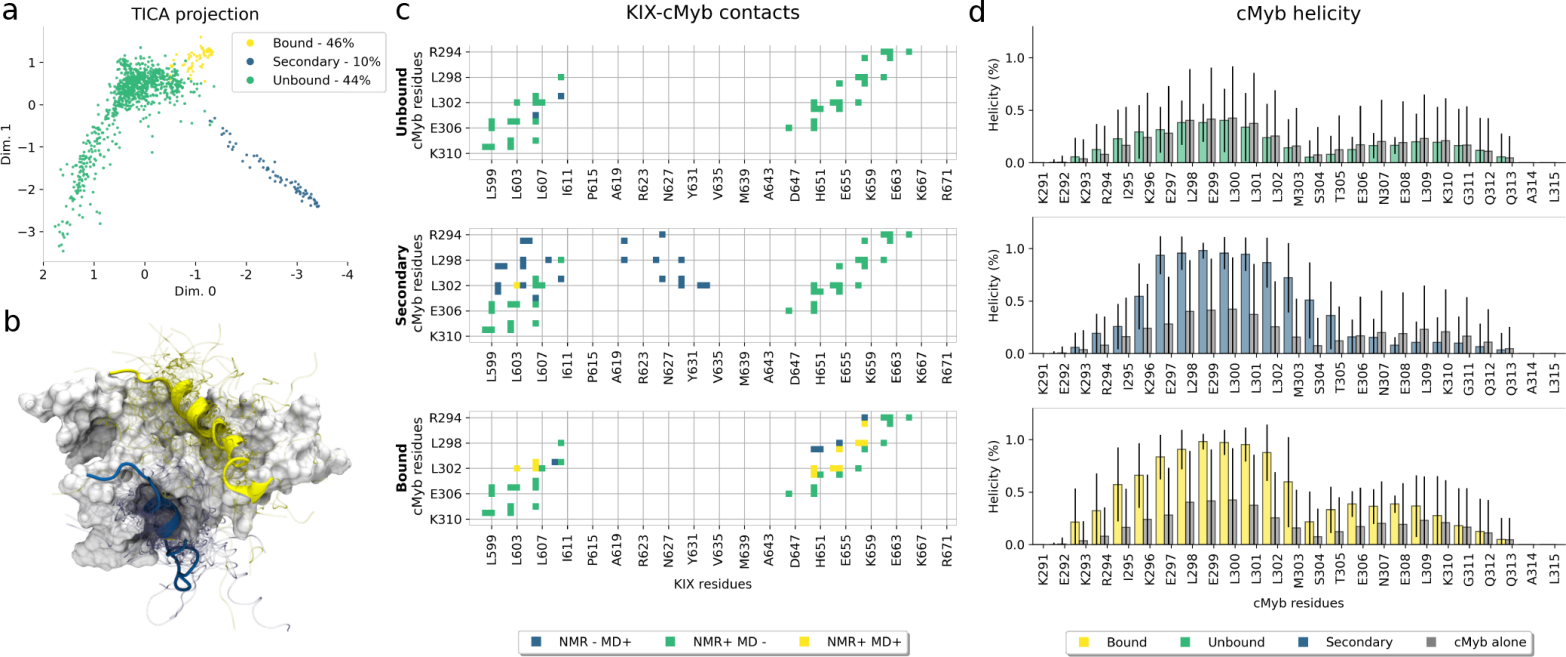
KIX and c-Myb binding model. (a) States distribution across the TICA space: microstates are represented as dots and are colored following their macrostate assignment. (b) Representative structures: PDB structure 1SB0 is depicted with KIX as a gray surface, c-Myb bound to the primary interface as a yellow ribbon, and c-Myb bound to the secondary interface as a blue ribbon. c-Myb backbones for 30 representative MD structures of *bound* and *secondary* states are displayed with blurry yellow and blue clouds, respectively. (c) Macrostate contact fingerprint: profile of contacts established between c-Myb and KIX in each macrostate in at least 50% of the structures. Blue color represents contacts present in the state but not in the original NMR structure; green indicates original NMR contacts not found in the MSM state; and yellow squares represent contact matches found in both NMR and MD structures. (d) Macrostate cMyb helicity: helicity fraction per residue of c-Myb in each macrostate. Helicity for the cMyb peptide alone is depicted in gray in each plot for comparison.

The full data set of the AdaptiveBandit run accounted for a total simulation time of ∼450 μs, split across 40 epochs, and was the one used to study the molecular features of KIX—c-Myb binding-and-folding. For the analysis, a slightly different MSM was built based on all-pair *C*_α_ + *C*_β_ distances between KIX and c-Myb, self-distances between *C*_α_ of c-Myb, secondary structure of c-Myb, and RMSD to the NMR bound conformation (PDB ID: 1SB0). The MSM defines three kinetically similar sets of conformations, referred to as macrostates (Figure 2.a and Figure S1.b): a highly populated state with an heterogeneous mixture of conformations (*unbound*), a well-defined c-Myb bound state (*bound*), and finally, a secondary bound state (*secondary*). Representative structures of all states can be found in Figure 2.b. The *bound* macrostate contains structures with a minimum RMSD of approximately 3 Å, with respect to the NMR structure. Complete binding and folding trajectory videos can be found in Table S1, with reconstructed trajectories from different epochs containing unique paths to the bound state (with RMSD < 4 Å with respect to the bound NMR structure).

The *bound* state identifies the primary cMyb bound pose in the hydrophobic groove between α-1 and α-3 of KIX. On average, it shares 36% of the fraction of native intermolecular contacts (*Q*_int_) with the original NMR structure, as shown in Figure 2.c. These contacts mainly involve the interaction of c-Myb residues Leu298 and Leu302 with residues across the primary binding interface: Leu302 contacts Leu603, Leu653, and specially Leu607 of KIX, which is buried down in the pocket, whereas Leu298 establishes additional native contacts with Ala610, Ile657, and Tyr658. *Q*_int_ reaches up to 80% in those microstates exhibiting the tightest bound conformations, and in addition to the leucine binding, they feature most of the contacts between the C-terminal half of c-Myb and KIX, which are not that prevalent across the *bound* macrostate (Figure S2). The missing main contacts account for the electrostatic interactions established between Arg294 and the region on α-3. There are some conformations where these interactions occur, but their prevalence in those microstates is less than 50%.

The secondary structure profile for MD-derived states matches the experimental description of c-Myb,^24,29^ as shown in Figure S3: the 25 residues are separated in two halves by residues Met303 and Ser304. The N-terminal half shows a high helical tendency, around 20–30% for residues in positions 297 to 302 with c-Myb in isolation, being maximal in bound states. Experimentally, this N-terminal half in isolation reaches even higher helicity levels (∼70%) when using an extended construct of c-Myb.^24^ On the other hand, the C-terminal section exhibits low helical propensity when in isolation and increases when bound to KIX. The full helix conformation only appears in those microstates with the tightest bound conformations.

### Secondary Binding Mode

The existence of alternative binding poses between c-Myb and KIX has also been reported.^24^ The MSM shows the presence of a secondary binding mode (termed *secondary*), occupying a novel interface, located between α-1 and α-2 (Figure 1.b and Figure S8). The interaction of the *secondary* state resembles the *bound* binding mode: the N-terminal half is folded in the typical α-helix, while the C-terminal section remains mostly unstructured. The presence of a native contact in this secondary binding mode is due to the penetration of Leu302, located close to Leu603’s backbone in KIX, rather than by side-chain proximity. Leu298 and Leu302 of c-Myb are deeply buried in a hydrophobic pocket composed of residues Val604, Val608, and Leu620 (found in the G2 helix, which connects α-1 and α-2) and Val629. Kinetically, there is a 10-fold difference in the mean first passage time for binding between both sites—(9.96 ± 3.57) × 10^3^ ns for binding to the *bound* site and (1.05 ± 0.46) × 10^5^ ns for the *secondary* site—that may account for the preferential binding of c-Myb to the primary interface.

### Model Validation

To validate the model, we compared the kinetic parameters derived from it with available information.^27^ Experimental values from Shammas et al. were calculated at temperatures ranging from 278 to 298 K, while simulations were executed at physiological temperatures (310 K). *k*_on_ values display a temperature independent tendency, whereas for temperature dependent variables, *k*_off_ and *k*_d_ values had to be extrapolated to 310 K (Figure S4). Hence, reference values for *k*_off_ and free energy (obtained from *k*_d_) resulted in 866 s^–1^ and −6.81 kcal mol^–1^, respectively.

Due to the size of the peptide compared to the solvation box, it is hard for the MSM to automatically define the correct bulk state. We, therefore, manually defined a bulk state that contains conformations where the distance between KIX and cMyb is maximized. The bulk state was defined by taking those microstates in which the minimum distance between KIX and cMyb is higher than a threshold. Consequently, some kinetic and thermodynamic estimates have a dependency on such distance threshold (Figure S6a) as this affects the definition of the bulk state. However, the computed *k*_off_ and free energy estimates are practically stable after a minimum separation distance of just 4 Å.

The obtained MSM estimations of *k*_on_ go between (2.72) × 10^7^ M^–1^ s^–1^ and (3.65) × 10^7^ M^–1^ s^–1^, in agreement with the experimental value (2.2 ± 0.2) × 10^7^ M^–1^ s^–1^.^27^*k*_off_ estimates range from 3.50 × 10^3^ s^–1^ to 21.70 × 10^3^ s^–1^, overestimating the extrapolated experimental value by an order of magnitude. Free energy estimates range between −7.35 kcal mol^–1^ and −6.27 kcal mol^–1^ depending on the choices of the analysis parameters, with the extrapolated experimental value inside this interval.

We further verified the reproducibility of kinetic and thermodynamic measurements to ensure model convergence by building multiple MSMs by using incrementally more trajectories. Convergence is reached at 150 μs on all the previous estimates (Figure S5). The discrepancy between the experimental reference and computed *k*_off_ values translates to a faster dissociation in our model. We also verified if this was due to normal discretization errors in the MSM projection or to the fact that our simulations did not obtain a complete bound conformation between KIX and cMyb. In order to test this hypothesis, additional long trajectories (8 replicas of 2 μs each) were run starting from bound NMR and MD-derived conformations. We constructed an MSM using both simulation data sets. However, the free energy estimations are only marginally improved (Figure S6b). Thus, we concluded that the additional bound simulations do not add additional information, and we restrict the analysis to just the AdaptiveBandit set of simulations as this is the most general case where no NMR information is available. Structural details of the bound state obtained from the 2 μs long runs can be seen in Figure S9, where we can observe that two of those replicas present an helicity reduction, corresponding to the unfolding of the C-term of cMyb while still being bound to KIX.

### Binding Follows Both Induced-Fit and Conformational Selection

**Figure 3:**
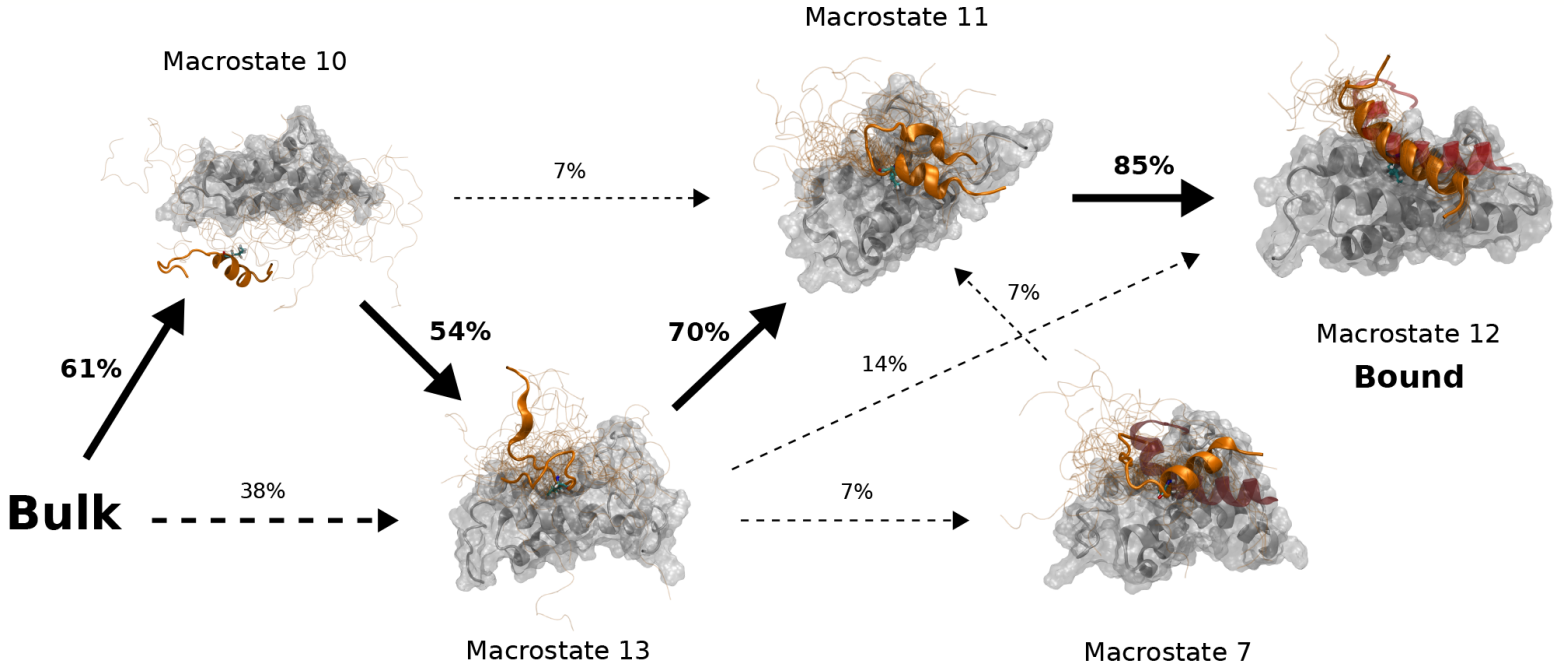
Complete c-Myb binding process to the KIX domain. Main pathways leading from *Bulk* (macrostate 14) to the *Bound* state (macrostate 12). Fluxes are shown as percentages near the arrows. Only those fluxes higher than 5% are shown. The arrow thickness is proportional to the flux percentage. Straight arrows indicate the maximum flux path, while dashed arrows show other fluxes. Each macrostate structure shows KIX as the white surface and ribbons and c-Myb as the orange ribbon and tubes. For each macrostate, 25 conformations are shown as thin tubes with one structure highlighted as a ribbon structure that includes the side chain of Leu302, colored by atom element. Additionally, for macrostates 7 and 12, the reference NMR c-Myb structure is shown as a transparent red ribbon for comparison.

In order to gain additional structural insight into the binding process, we constructed an MSM with a higher number of macrostates, using the same lag time. We used transition path theory^37,38^ to calculate fluxes leading from the purely bulk state to the bound conformations. Out of the 15 macrostates of the new MSM, only a reduced set of 6 is sufficient to explain binding to the primary interface (Figure S7). The other macrostates describe either the secondary binding mode or other unstable interactions between KIX and cMyb. The network generated by the flux interchanges between macrostates (Figure 3 and Figure S7) separates the binding and folding process into three events: the establishment of the initial contacts, binding and folding of the N-term section of cMyb, and finally, binding and folding of the C-term section of cMyb. The first step of the binding process features the first native contacts found across the KIX—c-Myb binding pathway, which involves residues Leu302 of c-Myb. The role of Leu302 as the main driving force for the interaction has already been described^3^ and is due in part to the kink in the helix created by neighboring residues Met303 and Ser304, which exposes Leu302 allowing for a deep penetration inside the binding pocket. Besides, one of the KIX residues contacted at this stage is Leu603, which is one of the most exposed residues in the hydrophobic pocket later occupied by Leu302.

**Figure 4:**
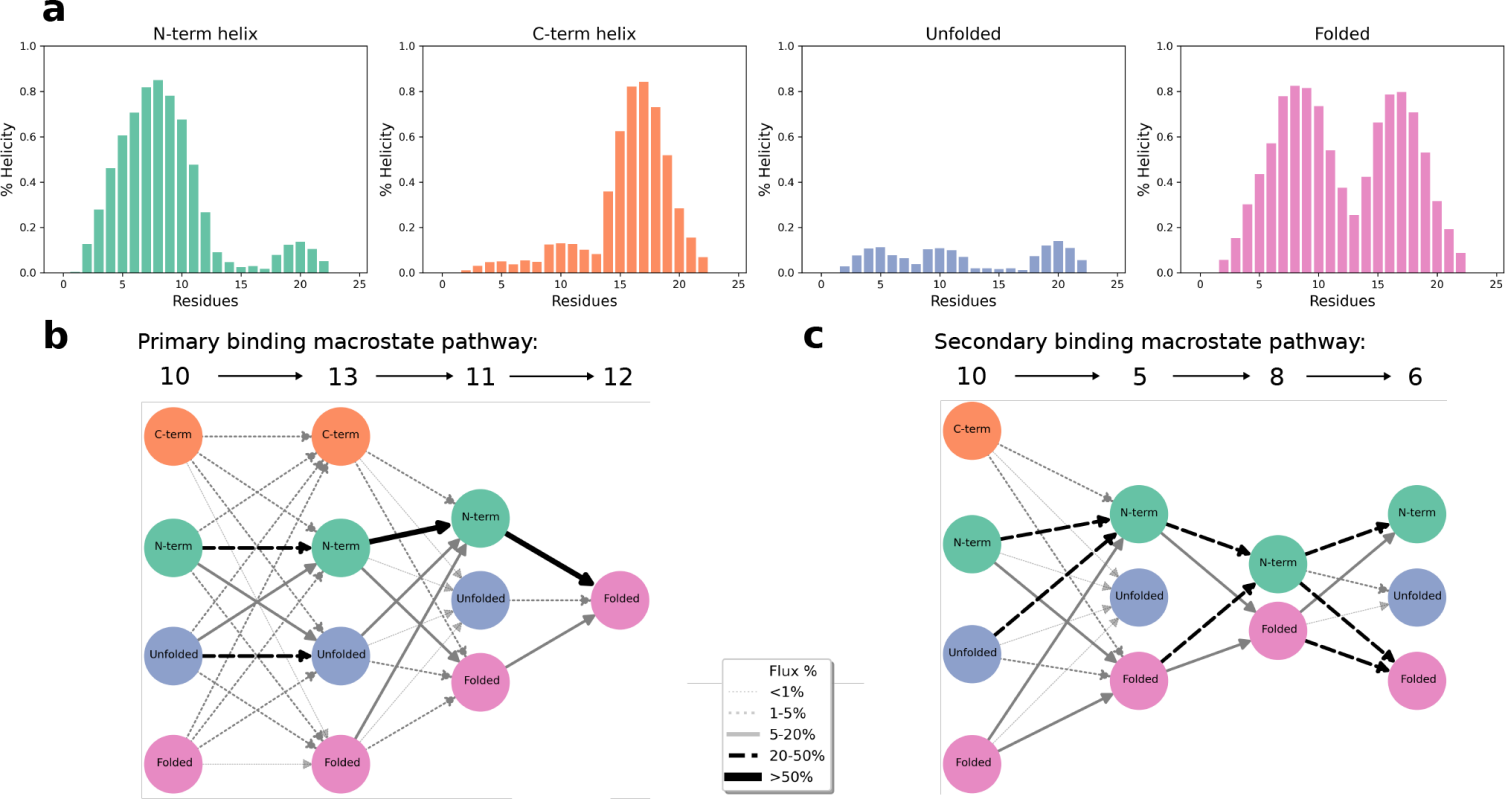
Detailed intramacrostate flux analysis for the main and secondary binding pathways. (a) Cluster centers corresponding to the four main cMyb folded states: N-terminal helix, C-terminal helix, unfolded, and folded helix. These centers were computed by using the mean helicity of all microstates. The centers are displayed with bar plots showing the helicity per residue. (b,c) Flux pathways for the main (b) and secondary (c) binding pathways. The selected macrostates were clustered using the four centers defined in (a), and the flux was recomputed using these newly defined clusters. Node colors and names indicate to which cluster center from (a) they correspond. Node positioning was manually set for visualization purposes. Arrows represent the connection between macrostates/clusters. Their color, thickness, and trace represent the percentage of the total flux traversing them.

To determine if cMyb folding precedes or follows binding at this step, we looked at the flux passing through different cMyb conformations in all of the macrostates (Figure 4b). We see that almost half of the flux goes through N-term to N-term helical conformations, suggesting that the presence of helix in residues 297 to 302 facilitates these first binding step, following a conformational selection mechanism. There is also a considerable flux going through unfolded to unfolded conformations, meaning Leu302 also binds through induced fit.

The second step of the binding process goes from macrostate 13 to macrostate 11, where several contacts are formed across the N-term of cMyb. The last step, which goes from macrostate 11 to 12 (the bound state), involves forming the last contacts on the C-term and completely folding cMyb. When looking at the flux through different cMyb conformations on these macrostates (Figure 4b), we see that almost all transitions from macrostate 13 to macrostate 12 go through states with some cMyb structure, with the majority of the flux going through N-term helical conformations until it reaches macrostate 12, where only fully folded conformations exist.

The overall mechanism works as an induced-fit binding and folding since the flux almost never goes through Folded to Folded transitions, but we can see a mixed mechanism during the first binding steps, where both conformational selection and induced-fit seem to take a part in facilitating the first contacts through cMyb’s Leu302. We can also see a similar effect for the secondary bound state (Figure 4c). The secondary binding mode occurs as a general induced-fit mechanism, but the overall flux to the bound state is heavily favored by N-term helical conformations. Interestingly, we can even see that the flux favors transitions from fully folded conformations to N-term helical conformations between macrostates 5 and 8, and also the secondary bound state, macrostate 6, contains different cMyb helical conformations.

In summary, initial steps can greatly benefit from prefolded helical structures of c-Myb (Figure 3), although *binding before folding* is also observed. The binding of helical conformations dominates the initial steps of the interaction, but for the interaction of the C-terminal tail, folding follows binding. No limiting steps in the binding process are observed; hence no possible transition states can be defined, as pointed out by experimental reports.^27^

## Conclusions

The analysis presented here provides a detailed molecular description of binding of c-Myb to the primary interface of KIX, summarized as a two-step process, where initially the N-terminal region of c-Myb binds with a preferred helical conformation, allowing the formation of native contacts and, in the last step, folding and binding of the C-terminal. Study of the fluxes derived from the MSM shows the relevance of residue Leu302, not only in the final bound structure but also as that responsible for establishing the first contacts and serving as an anchoring point between c-Myb and KIX.

The model describes an overall induced-fit binding mechanism, as complete folding of cMyb is only observed when native contacts have been formed. Conformational selection would affect only the first binding stage on residues 298 to 302 and not the whole length of the peptide, whereas the latter stages of binding follow an induced-fit mechanism.

Overall, our results provide a detailed mechanistic model for the binding of c-Myb to the primary interface of KIX, as well as showing the interaction with a secondary binding site by using unbiased full-atom MD simulations and MSM analysis. The novel MD sampling approach used in this work, AdaptiveBandit, played a crucial role in resolving this type of folding and binding process. The method is implemented and available in the HTMD python package.^34^ However, more algorithms can be derived within the same bandit framework. While here we choose the reward to be minus the free energy, other choices could optimize different costs, for example, improving the precision of the off-rate or optimizing sampling in the context of structure prediction.

## Methods

### Molecular Dynamics Simulations

In order to generate initial conformations for c-Myb (residues 291 to 315), we ran multiple parallel simulations. The peptide was solvated in a cubic water box with 64 Å sides with a NaCl concentration of 0.05 M. First, the peptide was simulated at 500 K for 120 ns to unfold the initial structure. Then, 200 systems were built by placing one random unstructured c-Myb conformation in conjunction with KIX in opposite corners of a 64 Å side cubic water box with a NaCl concentration of 0.05 M, resulting in a final protein concentration of ∼3.2 mM.

All systems were built using HTMD^34^ and simulated with ACEMD,^39^ CHARMM22* force field,^40^ and TIP3P water model.^41^ A Langevin integrator was used with a damping constant of 0.1 ps^–1^. The integration time step was set to 4 fs with heavy hydrogen atoms (scaled up to four times the hydrogen mass) and holonomic constraints on all hydrogen-heavy atom bond terms. Electrostatics were computed by using PME with a cutoff distance of 9 Å and grid spacing of 1 Å. After energy minimization, equilibration for all systems was done in an NPT ensemble at 303 K, 1 atm, with heavy atoms constrained at 1 kcal mol^–1^ Å^2^. Energy minimization was run for 500 steps and the mixture equilibrated for 2 ns.

Production runs of 250 ns were performed at 310 K using the distributed computing project GPUGrid,^42^ following an adaptive sampling strategy. The final data set included 1,809 trajectories of 250 ns, resulting in an aggregated simulation time of ∼450 μs. Additionally, a set of long MD runs was performed starting from bound structures. Four models of the NMR-determined structure and four random bound conformations were selected and equilibrated, as previously described. In total, 8 long trajectories of ∼2 μs each were generated.

### Markov State Model Analysis

The projected space used for building the MSM included four different featurizations: all pair C_α_ + C_β_ atom distances between KIX and c-Myb to account for the interaction between the two proteins, self-distances between every C_α_ of c-Myb and its secondary structure to monitor its conformation, and finally, RMSD of cMyb with respect to the NMR bound structure, aligning the system using only the KIX domain. TICA was used at a lag time τ = 20 ns (implied time scales are shown in Figure S1.a) for both the distance features and the secondary structure features, taking the 4 most relevant components from the distance features (both interdistances and cMyb self-distances) and the 3 most relevant components from the secondary structure features.

The 8-dimensional projected data were discretized into 2,000 clusters using the mini-batch k-means algorithm.^43^ The microstates defined in the MSM were coarse-grained into larger metastable macrostates by using PCCA++.^44^ For the estimation of kinetic values, the original MSM was modified by creating an additional macrostate, considered the *bulk* state for all subsequent calculations to obtain the kinetics of binding. The bulk state was created by taking those microstates where the minimum distance between KIX and cMyb was higher than a threshold. Error in kinetic measures was estimated by creating 50 independent MSMs using a random set containing 80% of the simulation data.

To obtain the kinetic pathway of binding and folding, we increased the number of macrostates in the MSM by using PCCA++ again. Fluxes between macros were estimated using transition path theory.^37,38^ For the intramacrostate flux analysis, we computed the mean helicity of cMyb for each microstate in it and clustered them into 4 main states which describe the peptide’s grade of helix formation. All analysis were performed with HTMD.^34^

### AdaptiveBandit Sampling

The multiarmed bandit problem is defined by , where an action  and  is a (stochastic) reward function. We choose γ = 0 for totally discounted rewards. The optimal policy  selects actions *a*_*t*_ in order to maximize the cumulative future rewards. The construction of an optimal selection strategy requires handling the exploration–exploitation problem. AdaptiveBandit relies on the UCB1 algorithm,^35^ defining an upper confidence bound for each action-value estimate based on the number of times an action has been picked and the total amount of actions taken
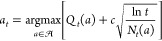
1where *t* denotes the total number of actions taken,  is the action-value estimation, *N*_*t*_(*a*) is the number of times action *a* has been selected (prior to time *t*), and *c* is a constant controlling the degree of exploration. As for the reward definition, there are different choices depending on the objective, e.g., here, the interest is sampling the bound metastable state, hence, we rewarded actions based on the stability of conformations using MSM estimations of the free energy for each state
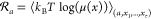
2where μ(*x*) is the equilibrium distribution estimated by the MSM with the currently available data and the average is performed over the frames in the trajectory starting from *a*. AdaptiveBandit uses the MSM discretized conformational space to define the action set and at each round acquires a random conformation from the selected states to respawn new simulations. A more formal description of the bandit framework and AdaptiveBandit in the context of adaptive sampling as well as analysis in simpler, analytical potentials is available at ref (32). The AdaptiveBandit sampling algorithm is made available in the HTMD^34^ Python package.

### Adaptive Sampling Parameters

For both the AdaptiveBandit and the count Adaptive runs, the construction of MSMs at each epoch was done using the residue–residue contacts between KIX and c-Myb measured as the minimum contacts between residues at a threshold of 5 Å and the backbone dihedral angles of c-Myb. Time independent component analysis (TICA)^45^ was used for dimensionality reduction using a lag time of τ = 20 frames and keeping the 3 first dimensions, which were later clustered with a k-centers algorithm. AdaptiveBandit was performed for 40 epochs with a *c* value of 0.01.

## Supporting Information Available

All supporting figures as well as the supporting table containing trajectories capturing the folding and binding of c-Myb with KIX domain (PDF)

## References

[ref1] Dyson H. J.; Wright P. E. Intrinsically unstructured proteins and their functions. Nat. Rev. Mol. Cell Biol. 2005, 6, 197 10.1038/nrm1589. 15738986

[ref2] Kussie P. H.; Gorina S.; Marechal V.; Elenbaas B.; Moreau J.; Levine A. J.; Pavletich N. P. Structure of the MDM2 oncoprotein bound to the p53 tumor suppressor transactivation domain. Science 1996, 274, 948–953. 10.1126/science.274.5289.948. 8875929

[ref3] Zor T.; De Guzman R. N.; Dyson H. J.; Wright P. E. Solution structure of the KIX domain of CBP bound to the transactivation domain of c-Myb. Journal of molecular biology 2004, 337, 521–534. 10.1016/j.jmb.2004.01.038. 15019774

[ref4] Buch I.; Giorgino T.; De Fabritiis G. Complete reconstruction of an enzyme-inhibitor binding process by molecular dynamics simulations. Proc. Natl. Acad. Sci. U. S. A. 2011, 108, 10184–10189. 10.1073/pnas.1103547108. 21646537 PMC3121846

[ref5] Plattner N.; Doerr S.; De Fabritiis G.; Noé F. Complete protein–protein association kinetics in atomic detail revealed by molecular dynamics simulations and Markov modelling. Nature Chem. 2017, 9, 1005 10.1038/nchem.2785. 28937668

[ref6] Borgia A.; Borgia M. B.; Bugge K.; Kissling V. M.; Heidarsson P. O.; Fernandes C. B.; Sottini A.; Soranno A.; Buholzer K. J.; Nettels D.; Kragelund B. B.; Best R. B.; Schuler B. Extreme disorder in an ultrahigh-affinity protein complex. Nature 2018, 555, 61 10.1038/nature25762. 29466338 PMC6264893

[ref7] Lindorff-Larsen K.; Piana S.; Dror R. O.; Shaw D. E. How fast-folding proteins fold. Science 2011, 334, 517–520. 10.1126/science.1208351. 22034434

[ref8] Piana S.; Lindorff-Larsen K.; Shaw D. E. Atomistic description of the folding of a dimeric protein. J. Phys. Chem. B 2013, 117, 12935–12942. 10.1021/jp4020993. 23882999

[ref9] Ganguly D.; Chen J. Atomistic details of the disordered states of KID and pKID. Implications in coupled binding and folding. J. Am. Chem. Soc. 2009, 131, 5214–5223. 10.1021/ja808999m. 19278259

[ref10] Ganguly D.; Zhang W.; Chen J. Electrostatically accelerated encounter and folding for facile recognition of intrinsically disordered proteins. PLoS Computational Biology 2013, 9, e1003363 10.1371/journal.pcbi.1003363. 24278008 PMC3836701

[ref11] Wang Y.; Chu X.; Longhi S.; Roche P.; Han W.; Wang E.; Wang J. Multiscaled exploration of coupled folding and binding of an intrinsically disordered molecular recognition element in measles virus nucleoprotein. Proc. Natl. Acad. Sci. U. S. A. 2013, 110, E3743–E3752. 10.1073/pnas.1308381110. 24043820 PMC3791790

[ref12] Best R. B.; Zheng W.; Mittal J. Balanced protein–water interactions improve properties of disordered proteins and non-specific protein association. J. Chem. Theory Comput. 2014, 10, 5113–5124. 10.1021/ct500569b. 25400522 PMC4230380

[ref13] Ganguly D.; Chen J. Modulation of the disordered conformational ensembles of the p53 transactivation domain by cancer-associated mutations. PLoS computational biology 2015, 11, e1004247 10.1371/journal.pcbi.1004247. 25897952 PMC4405366

[ref14] Hicks A.; Zhou H.-X. Temperature-induced collapse of a disordered peptide observed by three sampling methods in molecular dynamics simulations. J. Chem. Phys. 2018, 149, 072313 10.1063/1.5027409. 30134733 PMC5966312

[ref15] Dey S.; MacAinsh M.; Zhou H.-X. Sequence-dependent backbone dynamics of intrinsically disordered proteins. J. Chem. Theory Comput. 2022, 18, 6310–6323. 10.1021/acs.jctc.2c00328. 36084347 PMC9561007

[ref16] Zwier M. C.; Pratt A. J.; Adelman J. L.; Kaus J. W.; Zuckerman D. M.; Chong L. T. Efficient atomistic simulation of pathways and calculation of rate constants for a protein–peptide binding process: application to the MDM2 protein and an intrinsically disordered p53 peptide. journal of physical chemistry letters 2016, 7, 3440–3445. 10.1021/acs.jpclett.6b01502. 27532687 PMC5008990

[ref17] Zhou G.; Pantelopulos G. A.; Mukherjee S.; Voelz V. A. Bridging microscopic and macroscopic mechanisms of p53-MDM2 binding with kinetic network models. Biophysical journal 2017, 113, 785–793. 10.1016/j.bpj.2017.07.009. 28834715 PMC5567610

[ref18] Morrone J. A.; Perez A.; MacCallum J.; Dill K. A. Computed binding of peptides to proteins with MELD-accelerated molecular dynamics. J. Chem. Theory Comput. 2017, 13, 870–876. 10.1021/acs.jctc.6b00977. 28042966

[ref19] Paul F.; Wehmeyer C.; Abualrous E. T.; Wu H.; Crabtree M. D.; Schöneberg J.; Clarke J.; Freund C.; Weikl T. R.; Noé F. Protein-peptide association kinetics beyond the seconds timescale from atomistic simulations. Nat. Commun. 2017, 8, 1095 10.1038/s41467-017-01163-6. 29062047 PMC5653669

[ref20] Zou R.; Zhou Y.; Wang Y.; Kuang G.; Ågren H.; Wu J.; Tu Y. Free energy profile and kinetics of coupled folding and binding of the intrinsically disordered protein p53 with MDM2. J. Chem. Inf. Model. 2020, 60, 1551–1558. 10.1021/acs.jcim.9b00920. 32053358

[ref21] Zosel F.; Mercadante D.; Nettels D.; Schuler B. A proline switch explains kinetic heterogeneity in a coupled folding and binding reaction. Nat. Commun. 2018, 9, 3332 10.1038/s41467-018-05725-0. 30127362 PMC6102232

[ref22] Chong S.-H.; Im H.; Ham S. Explicit Characterization of the Free Energy Landscape of pKID–KIX Coupled Folding and Binding. ACS Central Science 2019, 5, 1342–1351. 10.1021/acscentsci.9b00200. 31482116 PMC6716127

[ref23] Robustelli P.; Piana S.; Shaw D. E. Mechanism of coupled folding-upon-binding of an intrinsically disordered protein. J. Am. Chem. Soc. 2020, 142, 11092–11101. 10.1021/jacs.0c03217. 32323533

[ref24] Arai M.; Sugase K.; Dyson H. J.; Wright P. E. Conformational propensities of intrinsically disordered proteins influence the mechanism of binding and folding. Proc. Natl. Acad. Sci. U. S. A. 2015, 112, 9614–9619. 10.1073/pnas.1512799112. 26195786 PMC4534220

[ref25] Giri R.; Morrone A.; Toto A.; Brunori M.; Gianni S. Structure of the transition state for the binding of c-Myb and KIX highlights an unexpected order for a disordered system. Proc. Natl. Acad. Sci. U. S. A. 2013, 110, 14942–14947. 10.1073/pnas.1307337110. 23980173 PMC3773806

[ref26] Gianni S.; Morrone A.; Giri R.; Brunori M. A folding-after-binding mechanism describes the recognition between the transactivation domain of c-Myb and the KIX domain of the CREB-binding protein. Biochemical and biophysical research communications 2012, 428, 205–209. 10.1016/j.bbrc.2012.09.112. 23026051

[ref27] Shammas S. L.; Travis A. J.; Clarke J. Remarkably fast coupled folding and binding of the intrinsically disordered transactivation domain of cMyb to CBP KIX. J. Phys. Chem. B 2013, 117, 13346–13356. 10.1021/jp404267e. 23875714 PMC3807845

[ref28] Toto A.; Camilloni C.; Giri R.; Brunori M.; Vendruscolo M.; Gianni S. Molecular recognition by templated folding of an intrinsically disordered protein. Sci. Rep. 2016, 6, 21994 10.1038/srep21994. 26912067 PMC4766501

[ref29] Poosapati A.; Gregory E.; Borcherds W. M.; Chemes L. B.; Daughdrill G. W. Uncoupling the folding and binding of an intrinsically disordered protein. Journal of molecular biology 2018, 430, 2389–2402. 10.1016/j.jmb.2018.05.045. 29890118 PMC6082395

[ref30] Shammas S. L.; Travis A. J.; Clarke J. Allostery within a transcription coactivator is predominantly mediated through dissociation rate constants. Proc. Natl. Acad. Sci. U. S. A. 2014, 111, 12055–12060. 10.1073/pnas.1405815111. 25092343 PMC4143058

[ref31] Sugase K.; Dyson H. J.; Wright P. E. Mechanism of coupled folding and binding of an intrinsically disordered protein. Nature 2007, 447, 1021 10.1038/nature05858. 17522630

[ref32] Pérez A.; Herrera-Nieto P.; Doerr S.; De Fabritiis G. AdaptiveBandit: a multi-armed bandit framework for adaptive sampling in molecular simulations. J. Chem. Theory Comput. 2020, 16, 4685–4693. 10.1021/acs.jctc.0c00205. 32539384

[ref33] Doerr S.; De Fabritiis G. On-the-fly learning and sampling of ligand binding by high-throughput molecular simulations. J. Chem. Theory Comput. 2014, 10, 2064–2069. 10.1021/ct400919u. 26580533

[ref34] Doerr S.; Harvey M.; Noé F.; De Fabritiis G. HTMD: high-throughput molecular dynamics for molecular discovery. J. Chem. Theory Comput. 2016, 12, 1845–1852. 10.1021/acs.jctc.6b00049. 26949976

[ref35] Auer P. Using confidence bounds for exploitation-exploration trade-offs. Journal of Machine Learning Research 2002, 3, 397–422.

[ref36] Prinz J.-H.; Wu H.; Sarich M.; Keller B.; Senne M.; Held M.; Chodera J. D.; Schütte C.; Noé F. Markov models of molecular kinetics: Generation and validation. J. Chem. Phys. 2011, 134, 174105 10.1063/1.3565032. 21548671

[ref37] Weinan E.; Vanden-Eijnden E. Towards a theory of transition paths. Journal of statistical physics 2006, 123, 503 10.1007/s10955-005-9003-9.

[ref38] Noé F.; Schütte C.; Vanden-Eijnden E.; Reich L.; Weikl T. R. Constructing the equilibrium ensemble of folding pathways from short off-equilibrium simulations. Proc. Natl. Acad. Sci. U. S. A. 2009, 106, 19011–19016. 10.1073/pnas.0905466106. 19887634 PMC2772816

[ref39] Harvey M. J.; Giupponi G.; Fabritiis G. D. ACEMD: accelerating biomolecular dynamics in the microsecond time scale. J. Chem. Theory Comput. 2009, 5, 1632–1639. 10.1021/ct9000685. 26609855

[ref40] Piana S.; Lindorff-Larsen K.; Shaw D. E. How robust are protein folding simulations with respect to force field parameterization?. Biophysical journal 2011, 100, L47–L49. 10.1016/j.bpj.2011.03.051. 21539772 PMC3149239

[ref41] Jorgensen W. L.; Chandrasekhar J.; Madura J. D.; Impey R. W.; Klein M. L. Comparison of simple potential functions for simulating liquid water. J. Chem. Phys. 1983, 79, 926–935. 10.1063/1.445869.

[ref42] Buch I.; Harvey M. J.; Giorgino T.; Anderson D. P.; De Fabritiis G. High-throughput all-atom molecular dynamics simulations using distributed computing. J. Chem. Inf. Model. 2010, 50, 397–403. 10.1021/ci900455r. 20199097

[ref43] Pedregosa F.; Varoquaux G.; Gramfort A.; Michel V.; Thirion B.; Grisel O.; Blondel M.; Prettenhofer P.; Weiss R.; Dubourg V.; Vanderplas J.; Passos A.; Cournapeau D.; Brucher M.; Perrot M.; Duchesnay E. Scikit-learn: Machine learning in Python. Journal of machine learning research 2011, 12, 2825–2830.

[ref44] Röblitz S.; Weber M. Fuzzy spectral clustering by PCCA+: application to Markov state models and data classification. Advances in Data Analysis and Classification 2013, 7, 147–179. 10.1007/s11634-013-0134-6.

[ref45] Pérez-Hernández G.; Paul F.; Giorgino T.; De Fabritiis G.; Noé F. Identification of slow molecular order parameters for Markov model construction. J. Chem. Phys. 2013, 139, 015102 10.1063/1.4811489. 23822324

