# Supplementary material for: Binding-and-Folding Recognition of an Intrinsically Disordered Protein Using Online Learning Molecular Dynamics
Source: J Chem Theory Comput. 2023 Jun 21;19(13):3817–24. doi: 10.1021/acs.jctc.3c00008 (PMC10863933; doi:10.1021/acs.jctc.3c00008)
Supplement: Supplementary file 1 — ct3c00008_si_001.pdf [file ct3c00008_si_001.pdf]

Supporting Information: Binding-and-folding recognition of an intrinsically disordered protein using online learning molecular dynamics

Pablo Herrera-Nieto,[†,‖] Adrià Pérez ,[†,‡,‖] and Gianni De Fabritiis[*,†,¶,§]

†*Computational Science Laboratory, Universitat Pompeu Fabra, Barcelona Biomedical Research Park (PRBB), C Dr. Aiguader 88, 08003, Barcelona, Spain*

‡*Acellera Labs, C Dr Trueta 183, 08005, Barcelona, Spain*

¶*Acellera Ltd, Devonshire House 582, HA7 1JS, United Kingdom*

§*Institució Catalana de Recerca i Estudis Avançats (ICREA), Passeig Lluis Companys 23, 08010 Barcelona, Spain*

‖*These authors contributed equally to this work*

E-mail: gianni.defabritiis@upf.edu

Table S1: **Macrostate statistics for the 15 macrostate MSM.** Table shows several structural metrics for each macrostate on the 15 macrostate model used to gain additional structural insights of the binding process. Columns show macrostate number, macrostate probability, minimum and mean RMSD of cMyb to the bound structure, maximum and mean helicity percentages , maximum and mean fraction of native binding contacts (FNBC) and minimum and mean RMSD of cMyb to the secondary bound structure, computed against a conformation extracted from macrostate 6. All mean values are shown with ± the standard deviation.

Macro	Macro prob(%)	Min RMSD(Å)	Mean RMSD(Å)	Max hel.(%)	Mean hel.(%)	Max FNBC	Mean FNBC	Min RMSD sec.(Å)	Min RMSD sec.(Å)
0	1.02	17.45	21.41 ± 1.06	0.44	0.06 ± 0.08	0.18	0.08 ± 0.03	9.46	25.11 ± 2.25
1	0.17	19.86	27.87 ± 2.51	0.56	0.06 ± 0.09	0.24	0.02 ± 0.03	1.59	20.99 ± 2.51
2	1.04	20.46	23.90 ± 1.23	0.76	0.38 ± 0.10	0.18	0.04 ± 0.05	6.19	18.98 ± 10.26
3	0.51	8.38	14.52 ± 2.08	0.60	0.04 ± 0.07	0.37	0.09 ± 0.05	19.18	25.60 ± 1.76
4	1.44	19.0	20.67 ± 0.46	0.68	0.37 ± 0.06	0.37	0.10 ± 0.04	12.32	24.50 ± 3.11
5	18.55	23.48	29.16 ± 1.67	0.76	0.27 ± 0.11	0.31	0.05 ± 0.05	2.18	13.80 ± 5.61
6	1.28	21.26	27.65 ± 1.35	0.84	0.36 ± 0.11	0.16	0.04 ± 0.02	0.00	4.29 ± 1.09
7	6.22	5.09	8.90 ± 1.61	0.72	0.38 ± 0.09	0.79	0.31 ± 0.13	19.15	22.72 ± 1.93
8	2.72	23.63	27.77 ± 1.71	0.80	0.36 ± 0.10	0.21	0.00 ± 0.01	0.95	7.04 ± 1.61
9	2.31	19.34	23.53 ± 1.21	0.68	0.33 ± 0.06	0.16	0.00 ± 0.01	3.21	12.54 ± 2.79
10	13.16	21.37	33.48 ± 6.21	0.80	0.13 ± 0.14	0.42	0.01 ± 0.03	0.91	26.98 ± 10.68
11	13.92	3.75	9.00 ± 1.68	0.72	0.32 ± 0.11	0.89	0.44 ± 0.10	18.90	24.11 ± 1.14
12	10.00	3.00	5.81 ± 0.99	0.84	0.52 ± 0.12	0.94	0.57 ± 0.13	20.92	23.80 ± 0.70
13	27.53	4.13	16.95 ± 5.41	0.80	0.14 ± 0.14	0.89	0.13 ± 0.10	3.02	26.32 ± 4.87
14 (Bulk)	0.13	55.11	66.41 ± 4.66	0.80	0.18 ± 0.16	0.00	0.00 ± 0.03	29.82	54.72 ± 8.62

Table S2: **Visualizations of representative trajectories showing the binding and folding of cMyb with the KIX domain.** The selected trajectories were reconstructed from different trajectory fragments spawned across different epochs in the adaptive sampling scheme. The seven trajectories listed represent the seven unique paths that sample conformations with RMSD lower than 4 Å to the bound pose.

Trajectory	Min RMSD (Å)	Trajectory length [ns]	Video URL
Trajectory 1	3.0	323.1	`https://youtu.be/XNCe88Yzxro`
Trajectory 2	3.1	631.7	`https://youtu.be/fZZji2cdaKI`
Trajectory 3	3.3	323.1	`https://youtu.be/6qYiyhsqtig`
Trajectory 4	3.5	706.5	`https://youtu.be/d8nvW8i_vHk`
Trajectory 5	3.7	521.7	`https://youtu.be/eAOnZcW3D5Y`
Trajectory 6	3.8	445.1	`https://youtu.be/vltn2zURsfY`
Trajectory 7	4.0	323.1	`https://youtu.be/Mn_wjOxmcvU`

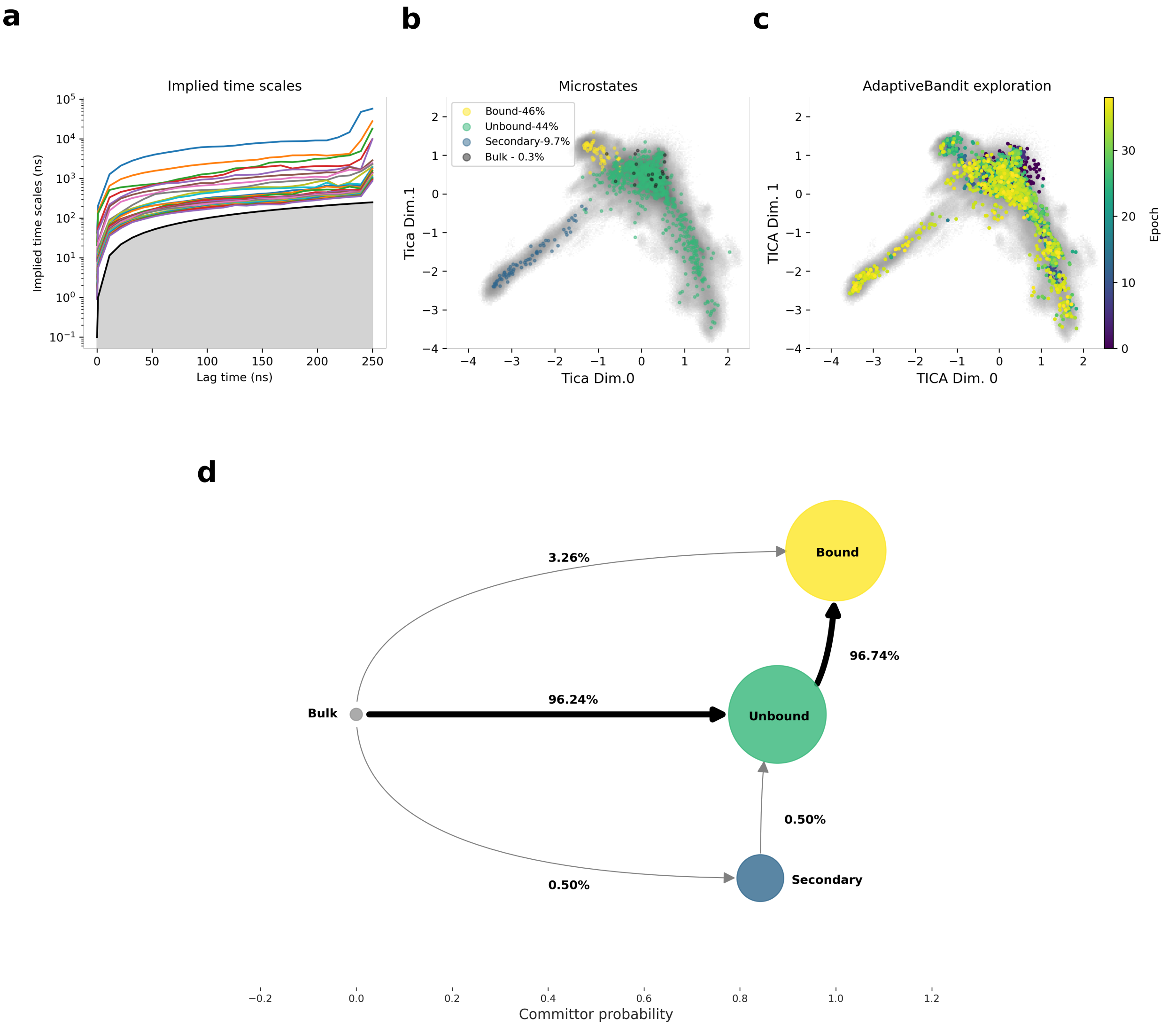

Figure S1: **Markov state model summary a)** Implied time scales of the MD data. **b)** Microstate distribution across the first two TICA dimensions. Each microstate is colored by its corresponding macrostate. The legend shows the population of each macrostate. **c)** AdaptiveBandit exploration of the TICA space. Each colored point indicates a starting point selected by AdaptiveBandit to respawn a new trajectory. The color indicates the epoch number. In grey, the area covered by the projected simulation data without clustering, both in b) and c). **d)** Flux pathway from *bulk* to *bound*. Nodes are placed according to the committor probability. The *y axis* is manually set for better visualization of the graph. Node size is proportional to the equilibrium distribution. Node color corresponds to macrostate assignment as in b). The flux percentage is shown near each arrow. The main pathway is indicated with black, thicker arrows.

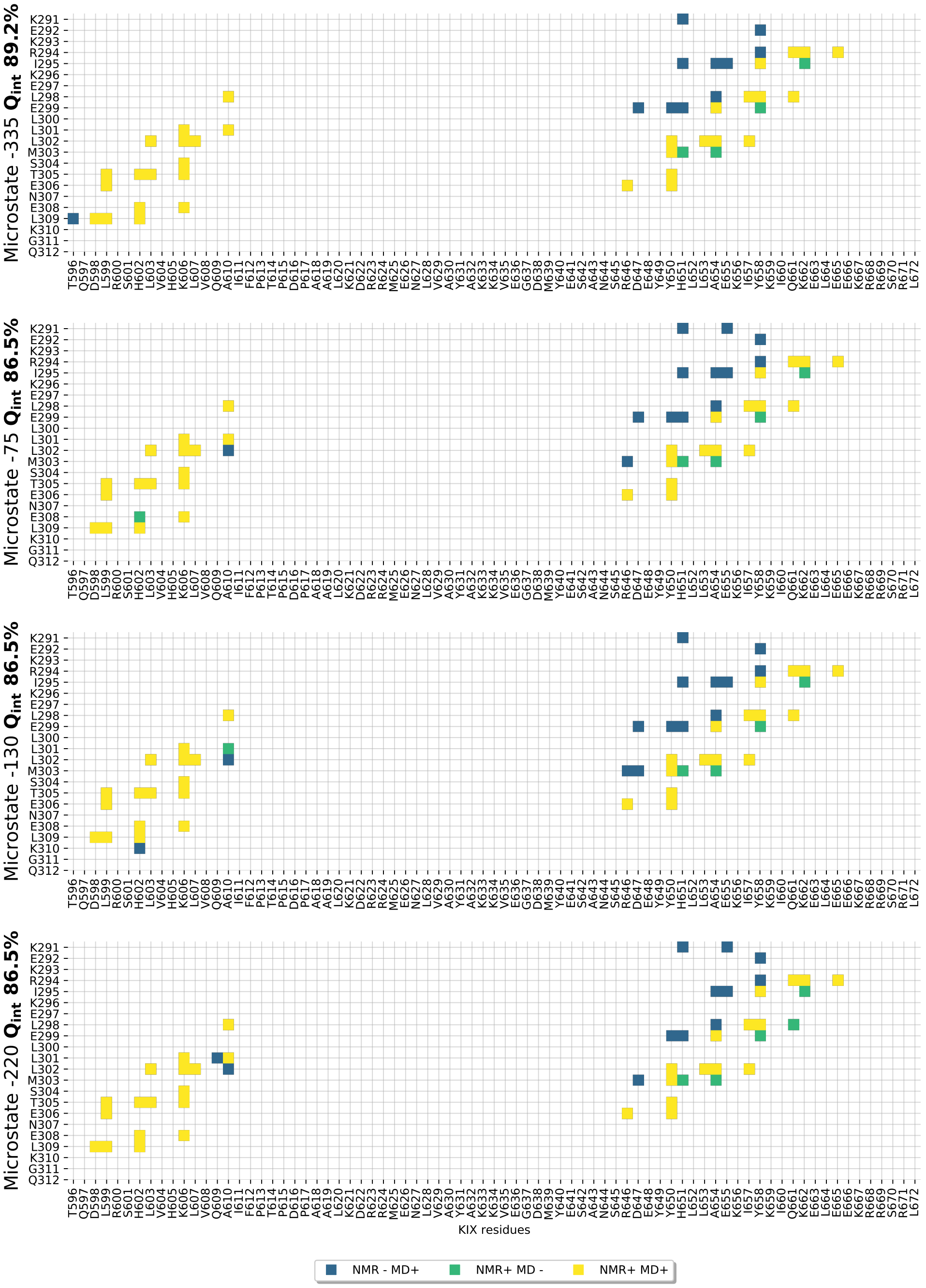

Figure S2: **Maximum Q_{int} microstates contact fingerprint.** Profile of contacts established between c-Myb and KIX in microstates with maximum fraction of native binding contacts Q_{int}. Blue color represents contacts present in the state but not in the original NMR conformation, green indicates native contacts not found in the MSM state and yellow squares represent a match on that contact, found in both the NMR model and MD microstate. A contact is considered present in a microstate when it appears in at least 50% of the conformations in that state.

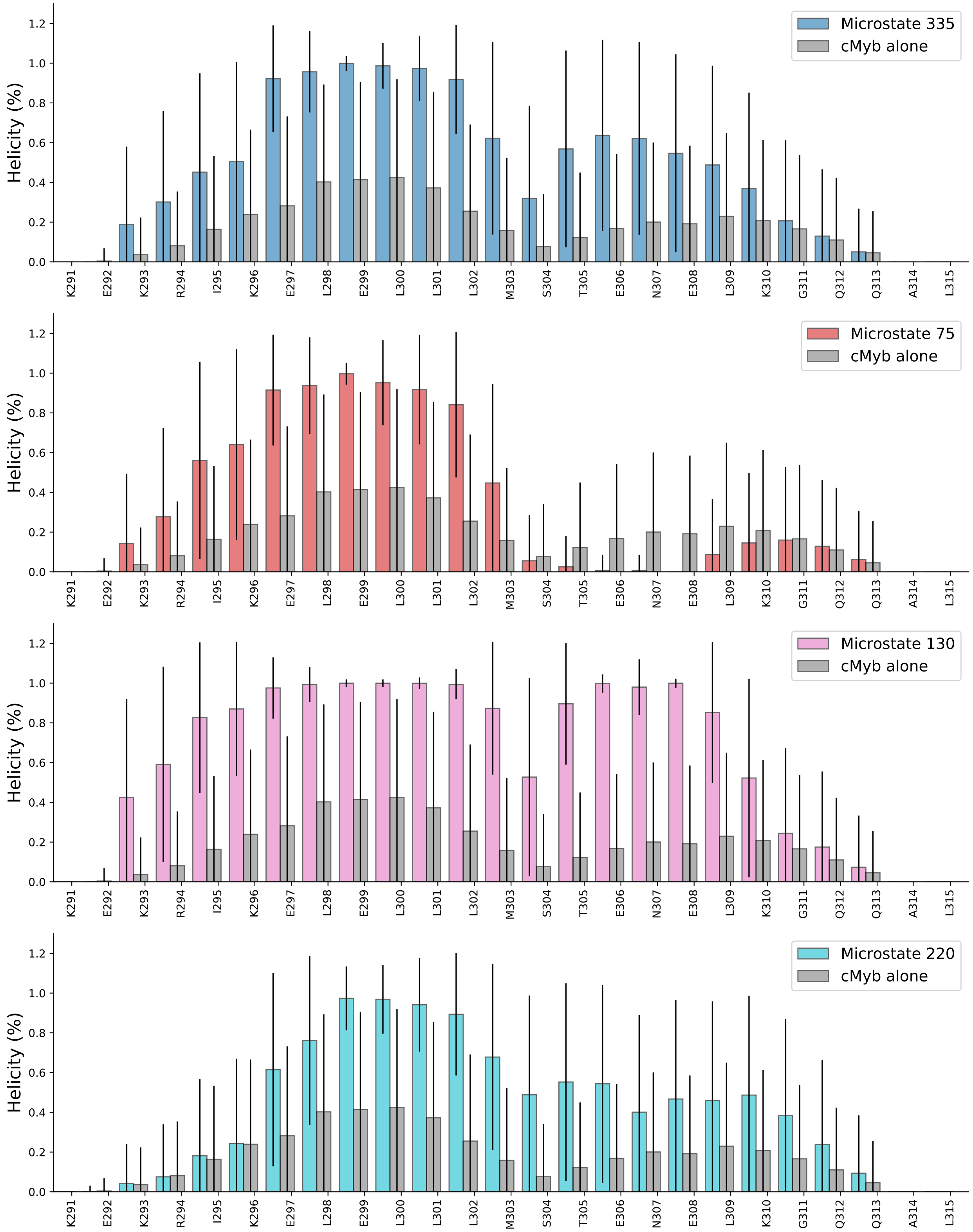

Figure S3: **c-Myb helicity.** Comparison of the by-residue helicity fraction of c-Myb between the four microstates with maximum Q_{int}. The helicity profile for the peptide in isolation is depicted in grey.

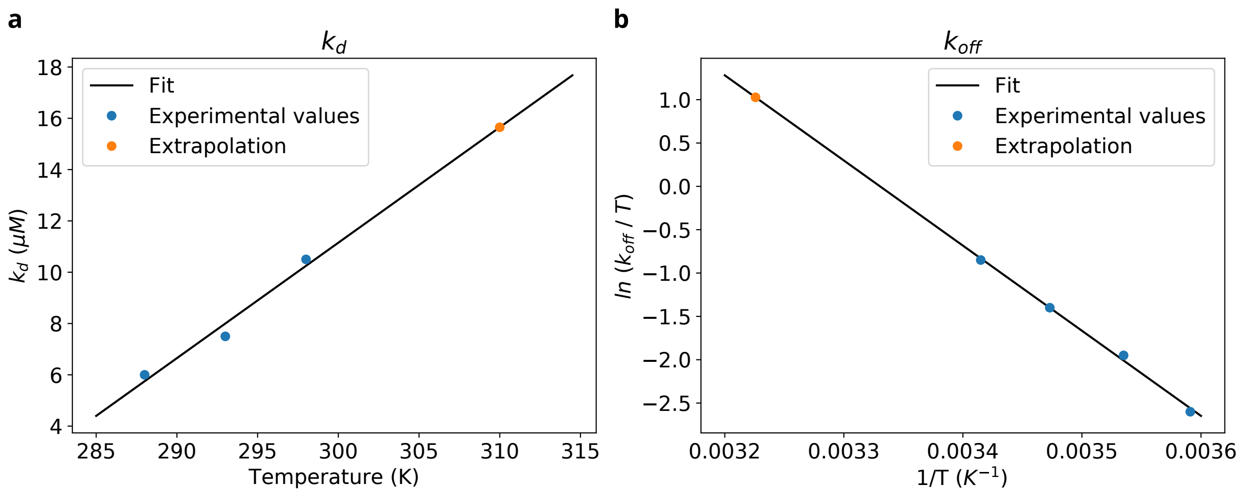

Figure S4: Extrapolations of **a)** k_d and **b)** k_{off} values from experimental data.

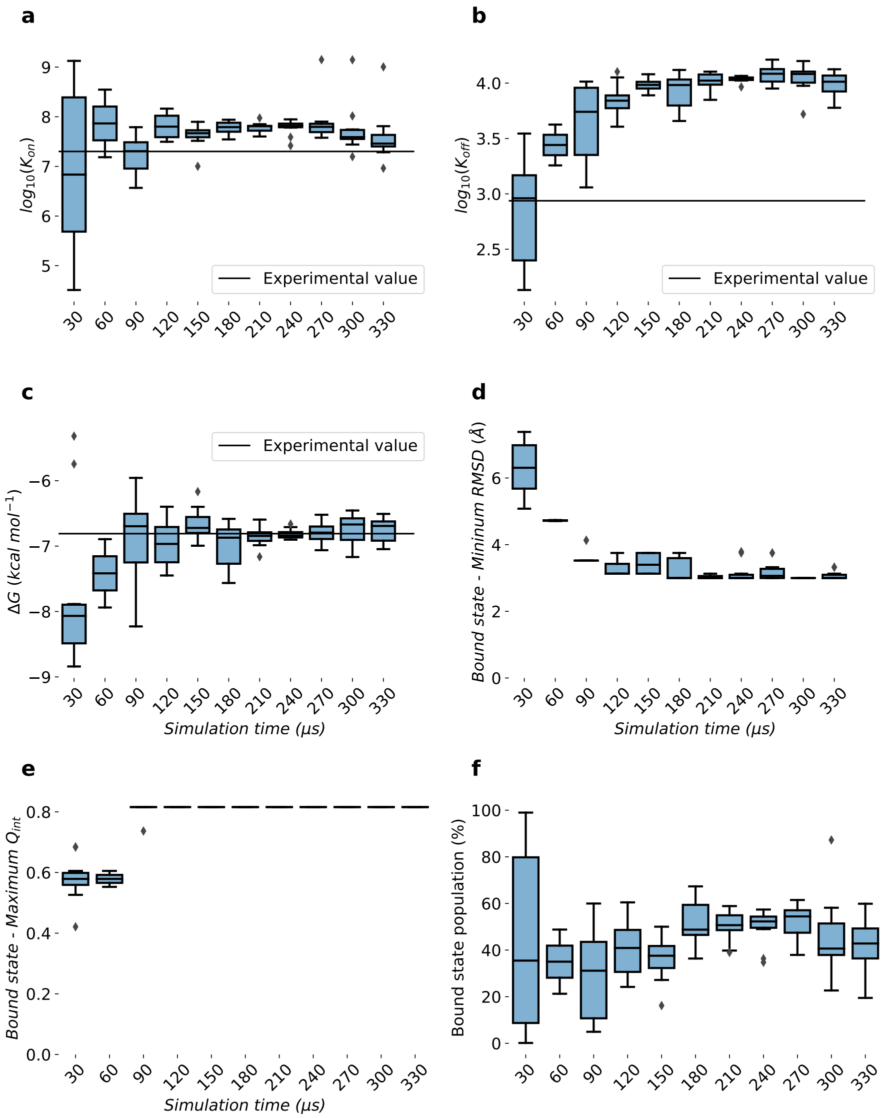

Figure S5: **Statistics convergence across the MD run** of **a)** k_{on}, **b)** k_{off}, **c)** free energy and **d)** microstate minimum RMSD, **e)** bound state maximum Q_{int} and **f)** bound state population, computed by the MSM. Each data point was calculated by building 10 different MSMs, bootstrapping 80% of the trajectories each time.

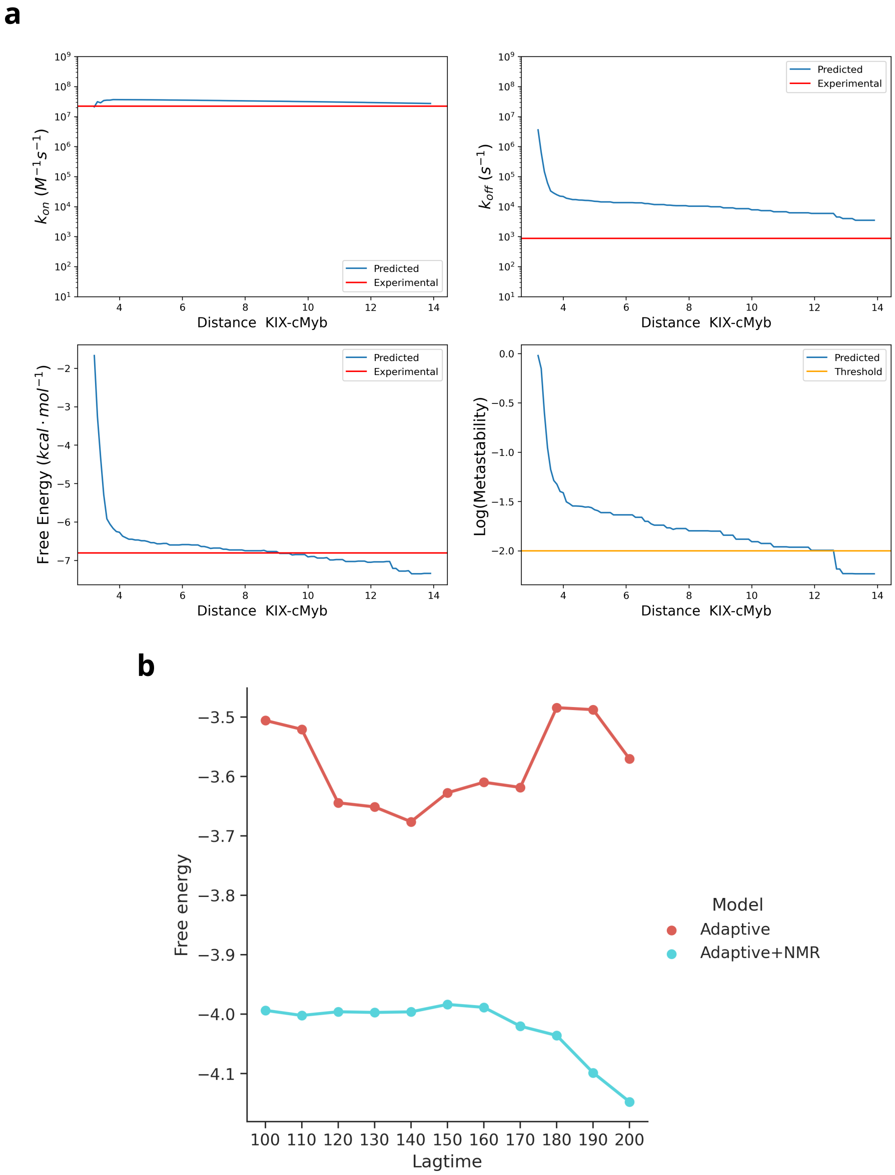

Figure S6: **a)** Bulk state variability of k_{on}, k_{off}, free energy and metastability term, depending on the maximum distance threshold between KIX and cMyb used. The metastability term is defined as the bulk state self-transition probability (named for plot simplicity). The blue line shows the variable estimate, the red line shows the reference experimental value and the yellow line shows the defined threshold for deciding whether the bulk state is stable enough or not. **b)** Free energy estimates at different MSM lag times for the AdaptiveBandit simulations alone and together with the long trajectories starting from the NMR structures. The models were performed without defining a bulk state.

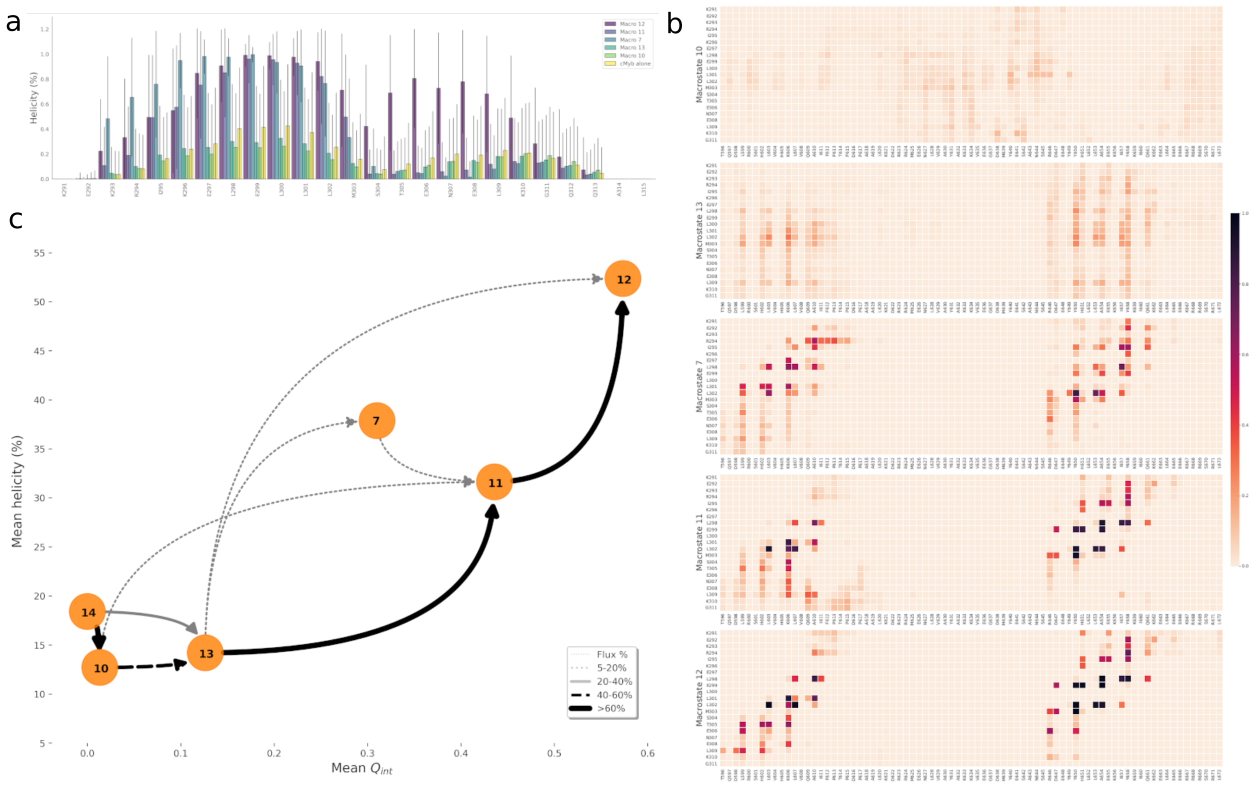

Figure S7: **Complete binding process of c-Myb to KIX.** Structural analysis of the states involved in the main binding flux pathway on the 15 macrostate MSM. **a)** Mean helicity per residue and **b)** mean contacts profile is shown for the macrostates present in the binding process. **c)** Main pathways leading from Macrostate 14 (*Bulk*) to Macrostate 12 (*Bound*). Nodes are placed according to the fraction of native contacts Q_{int} with respect to the NMR model on the x *axis*, and mean helicity on the y *axis*. Arrows represent the connection between macrostates, and their color, thickness and trace the percentage of the total flux traversing them.

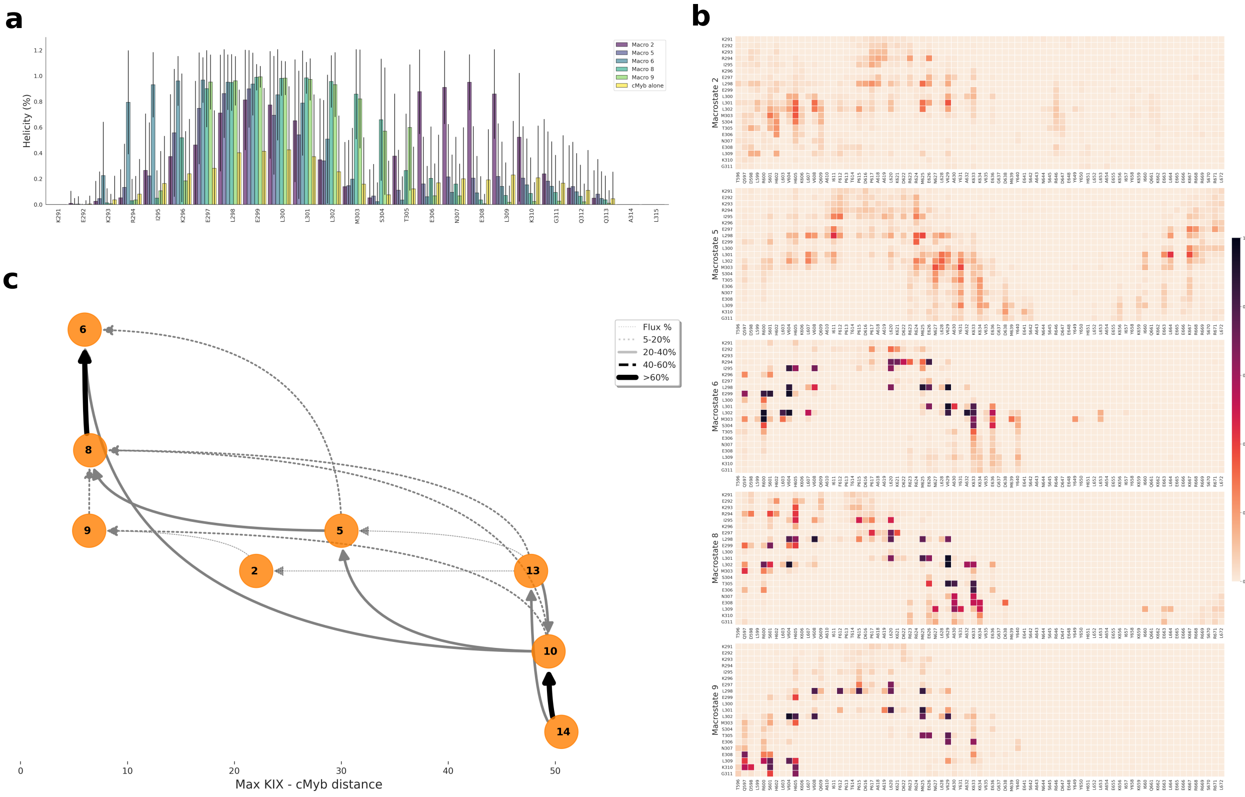

Figure S8: **Secondary binding path of KIX and c-Myb.** Study of the states involved in the secondary binding pathway, using the 15 macrostate MSM. For selected macrostates the **a)** mean helicity and **b)** KIX—c-Myb contacts profile is shown. Contact and helicity data for macrostates 10 and 13 are shown in FigS7. **c)** Main pathways leading from Macrostate 14 (*Bulk*) to Macrostate 6 (*Secondary*). Nodes are placed according to the maximum distance between KIX and cMyb of each state on the *x axis*. The *y axis* is manually set for better visualization of the graph. Arrows represent the connection between macrostates, and their color, thickness, and trace the percentage of the total flux traversing them.

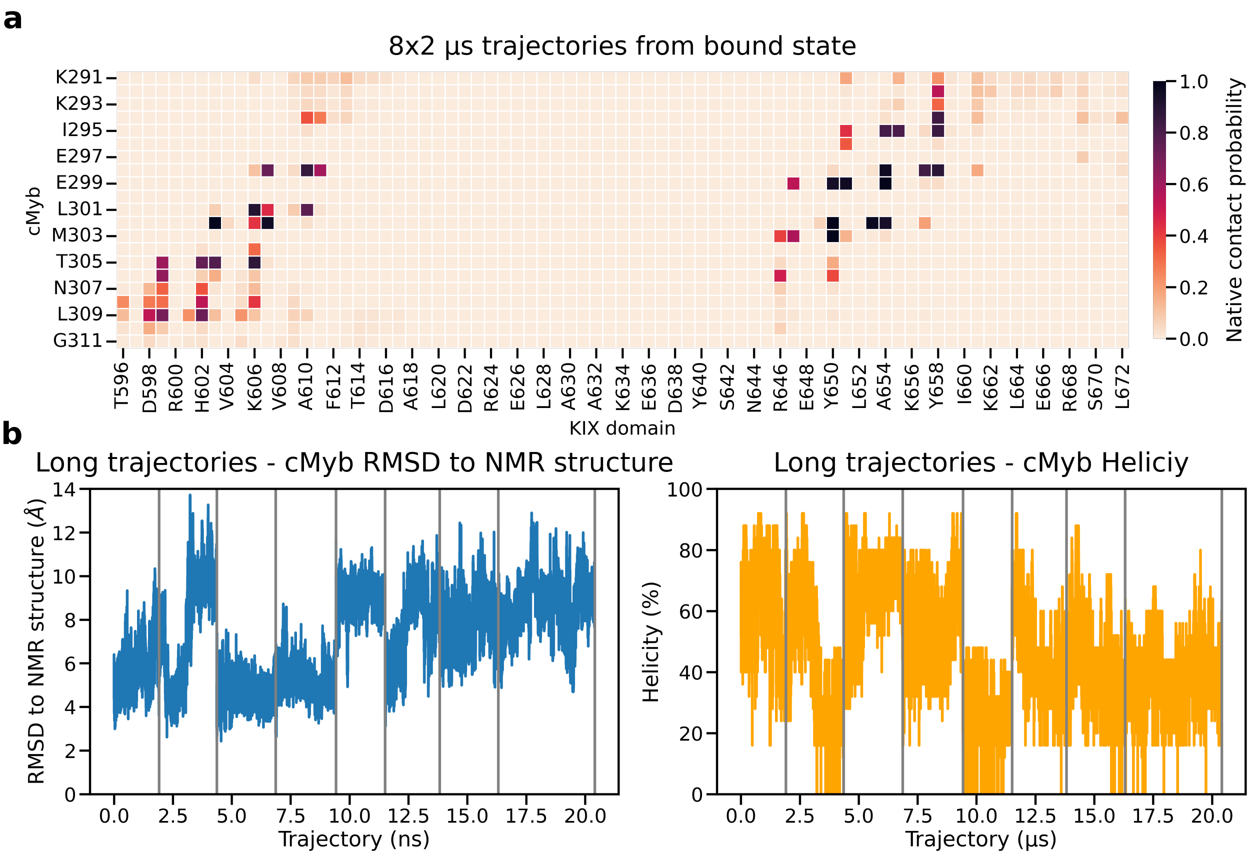

Figure S9: **Structural analysis of the 8 replicas of 2 μs trajectories.** Analysis of the 8 replicas of the long 2 μs trajectories performed starting from the bound NMR structure. **a)** Contact profile of all the trajectories, showing probabilities for all occurring contacts. **b)** Line plot of RMSD to NMR structure (left) and helicity percentage (right) across all trajectory frames. Vertical grey lines indicate separation between all the 8 replicas.
